# Retraction: Cryopreservation: A Review Article

**DOI:** 10.7759/cureus.r233

**Published:** 2026-07-10

**Authors:** Anurag N Jaiswal, Anjali Vagga

**Affiliations:** 1 Medical Education, Jawaharlal Nehru Medical College, Datta Meghe Institute of Medical Sciences, Wardha, IND; 2 Biochemistry, Jawaharlal Nehru Medical College, Datta Meghe Institute of Medical Sciences, Wardha, IND

This article has been retracted by the Editor-in-Chief due to plagiarism of the following article: Jang TH, Park SC, Yang JH, Kim JY, Seok JH, Park US, Choi CW, Lee SR, Han J. Cryopreservation and its clinical applications. Integr Med Res. 2017 Mar;6(1):12-18. doi: https://www.sciencedirect.com/science/article/pii/S221342201630155X?via%3Dihub 0.1016/j.imr.2016.12.001. Epub 2017 Jan 10. PMID: 28462139; PMCID: PMC5395684.

